# Emerging role of multi-detector computed tomography in the diagnosis of hematuria following percutaneous nephrolithotomy: A case scenario

**DOI:** 10.4103/0970-1591.56176

**Published:** 2009

**Authors:** S. E. Sivanandam, Georgie Mathew, Sanjay H. Bhat

**Affiliations:** Department of Urology, Amrita Institute of Medical Sciences, Kochi, India

**Keywords:** Angiography, embolization, spiral computed, therapeutic, tomography

## Abstract

Persistent hematuria is one of the most dreaded complications following percutanous nephrolithotomy (PCNL). Although invasive, a catheter-based angiogram is usually used to localize the bleeding vessel and subsequently embolize it. Advances in imaging technology have now made it possible to use a non invasive multi-detector computed tomography (MDCT) angiogram with 3-D reconstruction to establish the diagnosis. We report a case of post-PCNL hemorrhage due to a pseudo aneurysm that was missed by a conventional angiogram and subsequently detected on MDCT angiogram.

## INTRODUCTION

Traditionally, a conventional angiogram is the preferred investigation for hemorrhage following percutanous nephrolithotomy (PCNL). Although it has very high specificity to detect the source of bleeding,[1] it will not reveal the cause at all times. The fact that an angiogram may miss an organic lesion in a particular patient makes treatment planning difficult and delayed. Thus, there is a need for a further imaging study in this complex scenario.

## CASE REPORT

A 32-year-old male underwent a PCNL for a radio-opaque staghorn calculus in the left kidney. After access via a subcostal upper calyceal puncture, the tract was dilated up to 30 French size using Alkens coaxial dilators and complete clearance of the stone was achieved. Intra-operative blood loss was minimal, the immediate post-operative period was uneventful, and the patient was discharged on the third day after the operation. However, 15 days after the procedure, he was readmitted with a sudden onset of gross hematuria and clot retention. After endoscopic clot evacuation, the patient underwent a conventional renal angiogram that did not reveal any abnormality. Yet, persistent hematuria was observed for a week and his hemoglobin level decreased to 7 g/dl. As in the preoperative evaluation, the bleeding and coagulation profile continued to be normal and a repeat urine culture did not reveal any growth.

**Figure 1:**
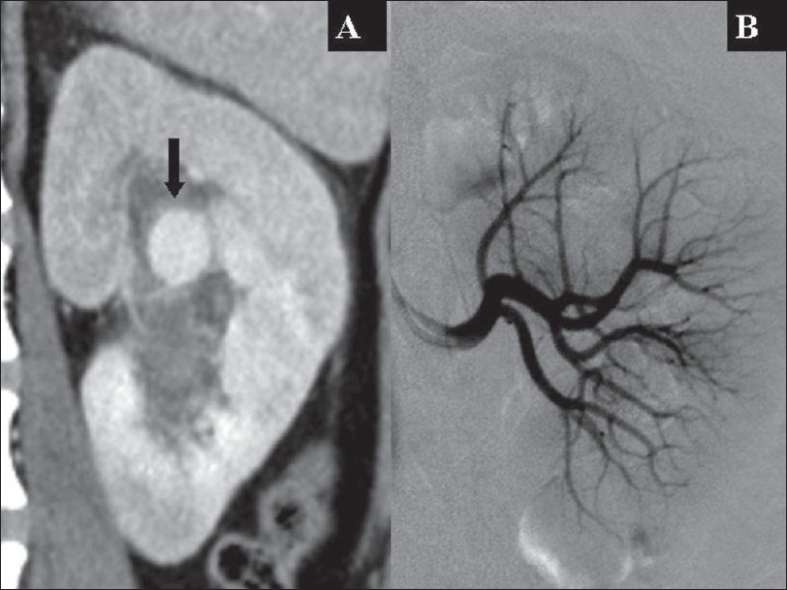
MDCT section (A) shows the interpolar pseudo aneurysm (block arrow) of the left kidney in comparison with a normal conventional angiogram (B)

**Figure 2:**
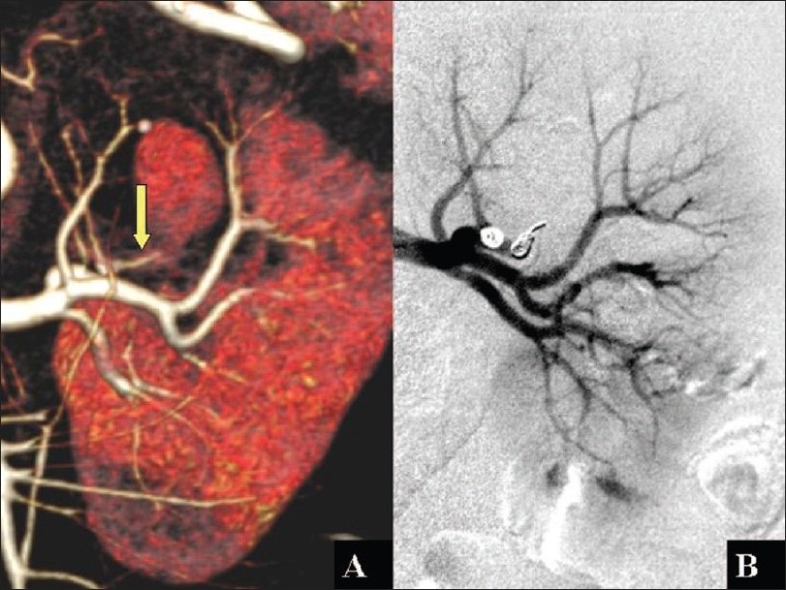
A 3-D reconstructed MDCT image (A) demonstrates the feeding vessel (yellow arrow) to the pseudo aneurysm branching from the posterior segmental renal artery and the selective embolization using metal coils with minimal parenchymal loss on a conventional angiogram (B)

In view of the persistent hematuria, he was submitted to a MDCT angiogram. After establishing access through an anterior cubital vein, around 90 ml (1–1.5 ml/kg) of non ionic iodinated contrast (Iohexol™-300 mg iodine/ml) was injected at a rate of 4.5 ml/min using a power injector followed by saline flush. The system was set to initiate the acquisition process after adequate opacification of the aorta using the bolus-triggering method. Image acquisition was done at 0.6 mm slice thickness using 64-slice MDCT (SIEMENS™). Reformatting of the axial sections into coronal and sagittal planes demonstrated an inter-polar pseudo aneurysm arising from a segmental renal arterial branch [Figure 1]. The feeding vessel to the pseudo aneurysm was mapped through 3-D reconstructed images by surface shaded display (SSD) and volume rendered technique and then successfully embolized by a conventional angiogram using platinum coated metal coils [Figure 2]. The patient stopped bleeding almost immediately after the procedure and a subsequent MDCT confirmed complete occlusion of the pseudo aneurysm. There have been no further episodes of hematuria and renal parameters were normal at the 6 month follow-up visit.

## DISCUSSION

Massive hematuria due to renovascular injury occurs in 0.3–1% of patients following PCNL.[2] The source of bleeding is usually an arteriovenous fistula (AVF) or a pseudo aneurysm, both of which can often be treated with selective embolization. Blood passage from the high pressure of the injured artery to the injured adjacent vein results in AVF and blood passage to the parenchyma leads to a pseudo aneurysm. The reported incidence of renal pseudo aneurysm following PCNL is 0.6–1%.[3] In less severe cases, bleeding arises from the venous channels or an infection of necrosed parenchymal tissue. Venous bleeding can usually be managed conservatively because the intrarenal venous system is quite resilient;[1] whereas, arterial bleeding needs to be embolized.

By convention, a selective angiogram has been used as a primary investigation when life-threatening bleeding occurs after PCNL. Although this gives the advantage of being able to perform an immediate therapeutic embolization, there may be limitations with respect to an accurate diagnosis. First, it may reveal the cause of hematuria only when there is active bleeding; intermittent or delayed bleeding, which usually occurs in pseudoaneurysms,[1] may be missed during the interval time. Secondly, venous bleeding and bleeding associated with infection may not be readily detected by conventional angiograms. Finally, being invasive in nature, there is an overall complication rate of 3.8% (1.3% major) due to vascular access for all diagnostic arteriographic procedures.[4]

The data for diagnostic accuracy of conventional angiogram in post-PCNL hemorrhage are limited; this is probably due to the low incidence of this complication even in a large series. In a series of 808 cases of PCNL, conventional angiography was used to accurately detect and subsequently embolize the cause of bleeding in 7 out of 8 cases of severe post-PCNL hemorrhage.[2] In another large series of PCNLs,[1] selective renal angiography could accurately detect pseudo aneurysms, AVF, and arterial lacerations with a sensitivity of 92.3% and specificity of 100%. However, it has to be noted that in both these series, bleeding patients with normal angiograms experienced spontaneous resolution of hematuria over a few days. In contrast, our patient was continuously bleeding due to a pseudo aneurysm that was missed on a conventional angiogram.

The impressive role of MDCT in identifying the source of acute gastrointestinal hemorrhage has driven the enthusiasm for its applications in other specialities.[5] Besides being non invasive and having short scanning times, MDCT provides thinner collimation, greater anatomic coverage, and better multi-planar reformatted images expanding its diagnostic role for various pathologic processes.[5] As opposed to angiography, MDCT offers information not only regarding the site of bleeding but also of the details regarding perinephric hematoma, residual stones, urinary extravasation, and hydronephrosis. It also allows improved interventional planning and lesion directed treatment. Since the diagnosis is being established prior to intervention, the total interventional time is reduced thereby decreasing the radiation exposure to the patient and the radiologist.[5] Though there are anecdotal reports of utilising MDCT in a post-PCNL scenario,[3] its efficacy needs to be evaluated in a larger cohort of patients and ideally in a randomised, controlled trial.

## CONCLUSION

Persistent post-PCNL hemorrhage is always a nightmare for an endourologist. Although a conventional angiogram is still the gold standard therapeutic procedure, it has certain limitations as a diagnostic modality. A MDCT scan is non invasive and has the potential to become a first-line investigation for post-PCNL hemorrhage.
